# Comparison of the caregivers’ and community health professionals’ views on home health care services for disabled older adults: a cross-sectional study in Beijing, China

**DOI:** 10.1186/s12913-021-06400-9

**Published:** 2021-04-26

**Authors:** Xiaojingyuan Xu, Chunyan Zhao, Meirong Wang, Xiaolei Chen, Shuang Shao, Juan Du

**Affiliations:** 1 School of General Practice and Continuing Education, Capital Medical University, Beijing, 100069 China; 2 Community Health Service Center, Beijing University of Posts and Telecommunications, Beijing, 100876 China

**Keywords:** Home health care services, Disabled older adults, Caregivers, Community health professionals

## Abstract

**Background:**

In an era of an increasingly ageing society, part of healthcare for older adults can be provided in patients’ homes, and the need for home health care services (HHCSs) is increasing. This study sought to determine whether a gap exists between the views of community health professionals and the caregivers of disabled older adults towards HHCSs in Beijing, China.

**Methods:**

A cross-sectional study with two comparative questionnaire surveys was conducted in Beijing, China. One survey was administered to the caregivers of disabled older adults, and the other was administered to health professionals in community health service institutions (CHSIs). T-tests and Wilcoxon signed-rank tests were used to explore potential differences between the views of caregivers and community health professionals towards HHCSs.

**Results:**

We received 370 valid questionnaires from caregivers and 224 questionnaires from health professionals. Of the 370 caregivers, 314 (84.9%) were willing to apply for HHCSs for the older adults, but only 20.5% (*N* = 76) received HHCSs. Over 80% of the caregivers accepted out-of-pocket costs less than 100 yuan per visit. Caregivers’ demands on home nursing services were significantly higher than those of health guidance services (*Z* = − 7.725, *P* < 0.001). Most of the 224 health professionals chose “health professionals’ personal safety cannot be guaranteed” as a problem limiting the provision of HHCSs (*N* = 151, 40.8%). The health professionals’ attitudes towards home nursing services were significantly less positive than those towards health guidance services (*Z* = − 10.081, *P* < 0.001). For home nursing services, health professionals’ attitude scores were lower than the caregivers’ demand scores (*Z* = − 4.960, *P* < 0.001), while for health guidance services, health professionals’ attitude scores were higher than the caregivers’ demand scores (*Z* = − 8.373, *P* < 0.001).

**Conclusions:**

Gaps exist between the views of caregivers and health professionals on HHCSs. Compared to health professionals with a higher willingness to provide health guidance services, caregivers need home nursing services. Feasible policies should be implemented to safeguard the rights and interests of health professionals, and qualified health professionals should be trained for HHCSs.

**Supplementary Information:**

The online version contains supplementary material available at 10.1186/s12913-021-06400-9.

## Background

China has the largest population in the world, with over 166 million people aged over 65 years and more than 23 million people aged over 80 years in 2018 [1]. The ageing process in China has led to the remarkably increasing prevalence and incidence of age-associated diseases [2]. The older adult population with disease-related disabilities exceeds 40 million [3]. Disease-related disability, especially physical disability and cognitive impairment, leads to dependency in activities of daily living (ADL), and the need for long-term care (LTC) is one of the consequences of disability and ADL dependency [4, 5]. Currently, LTC services are mainly provided by institutions (such as nursing homes and care centres) and families. Compared with institutional care services, home care services play a more fundamental role in the policy system of elderly care services in China [6]. Considering the high cost of institutional care services, e.g., the national average charge for disabled (including partially disabled) older adults was 2604 yuan per month and 5049 yuan per month in Beijing, approximately 80% of older adults chose home care services, while only less than 5% of them chose to seek care in institutions in 2016 [7]. In addition, Confucian teachings emphasize filial piety and intergenerational responsibility, and this Chinese culture places family members as the primary care providers for older people [8].

As a vital part of home care services, home health care services (HHCSs) are provided by general practitioners (GPs), nurses, physicians, social workers and other home care workers in many countries [9]. The providers come from agencies such as home health care organizations, social care organizations, community clinics, private agencies and general hospitals [10, 11]. Nurse specialists deliver nursing care, such as checking vital signs, exchanging various invasive tubes, changing dressings, collecting samples for examination, and administering medications and injections [12]. Other professionals provide physiotherapy, occupational or speech therapy, nutritional counselling and help with medical equipment or supplies during home visits [9]. They may also provide referral services, coordinating and managing services, education, training and counselling [12]. The fees for HHCSs are always partially covered by medical insurance [12, 13] or long-term care insurance (LTCI), which is designed to reimburse the cost of care associated with a disability [14, 15]. The governments in some countries, such as Finland, Turkey and the Netherlands, have established special legislation and regulations to govern home care services, including HHCSs [16–18]. In Jordan and Canada, HHCSs are not insured services, leading to chaotic management [9, 16].

The *“Guidelines on promoting the integration of eldercare services with medical care”* established in 2015 by the General Office of the State Council in China encouraged primary health care service institutions to provide HHCSs for older adults who are seriously ill, disabled and partially disabled [19]. Since then, many cities have begun to provide HHCSs for people of advanced age with a disability, no family in the residence and mobility limitations [20–23]. HHCSs include home nursing, home visit, health guidance and health counselling [19]. Most HHCSs can be delivered by health professionals in community health service institutions (CHSIs), such as GPs, community nurses, traditional Chinese medicine (TCM) practitioners, preventive care physicians and rehabilitation therapists. The Ministry of Human Resources and Social Security in China issued the *“Guidance on Pilot Cities to Launch Long-Term Care Insurance”* in June 2016, which signified the official initiation of LTCI in China. Fifteen cities were designated pilot cities [24]. LTCI, as a mechanism guaranteeing the implementation of HHCSs, covers the service forms of home care, nursing institution care and medical institution care. Life care and medical care are the two main core contents of LTCI. In general, the reimbursement ratio of LTCI ranges from 50 to 90%, according to the different economic levels and categories of cities [21–23].

As the capital city of China and a highly ageing city, Beijing has approximately 0.6 million disabled older adults, accounting for approximately 20% of the old-age population in this city [25]. The *“Regulations on the service of home care for the aged in Beijing”* established in 2015 stipulated eight types of home care services, such as housekeeping services, day care services, and medical services, and HHCSs for disabled older adults is one important part of these services [26]. *Dongcheng*, *Chaoyang*, *Haidian* and *Fengtai* were the four pilot districts in Beijing for home-based care for the aged since 2017. The status quo and views on HHCSs from both community health professionals and caregivers must be investigated to provide better HHCSs. The study aimed to determine whether a gap exists between the views of community health professionals and the caregivers of disabled older adults towards HHCSs.

## Methods

### Design

We conducted a cross-sectional study with a descriptive and comparative design. It consisted of two questionnaire surveys: one was administered to the caregivers of disabled older adults, and the other was administered to health professionals.

### Settings

As one of the six urban districts in Beijing (*Dongcheng, Xicheng, Haidian, Chaoyang, Fengtai*, and *Shijingshan*), *Haidian* district is located in the northwest urban area. It is divided into three regions according to the economic conditions, including the central, northern and western regions, which are similar to the capital functional core area (*Dongcheng* and *Xicheng*), the city functional expansion area (*Chaoyang, Fengtai*, and *Shijingshan*) and the urban development zone (*Tongzhou, Daxing, Shunyi, Changping*, and *Fangshan*), respectively. Therefore, *Haidian* District is considered representative of Beijing. Finally, of the 51 CHSIs in *Haidian* district, four from the central region, one from northern region and one from western region were selected according to the number of CHSIs and residents in these regions to include different economic situations.

### Participants

Caregivers of older adults who met all the criteria for older adults and him/herself were recruited when they came to CHSIs for medical help. Older adults should meet the following criteria: 1) aged 60 years or older and 2) living at home with at least one dependent ADL. The inclusion criteria for caregivers were as follows: 1) a family member or employed caregiver caring for older adults; 2) aged at least 18 years; 3) caring for older adults at home for at least 20 h per week for over 6 months; 4) no history of mental illness or cognitive impairment; and 5) able to communicate in Mandarin. According to the results in the study by Li Y conducted in the same area, the demand rate of HHCSs for disabled older adults was 70.3% [27], and thus the error tolerance in our study was set as 0.070. At least 325 caregivers were needed to adjust for an alpha error of 0.05 and a design effect of 2, and 380 older adults with 380 caregivers were eventually recruited.

Registered GPs, nurses, preventive care physicians, TCM practitioners, rehabilitation therapists and other workers in the selected CHSIs who had provided HHCSs in the past year were eligible for the survey of health professionals. Considering the willingness rate of health professionals to provide HHCSs as 50%, an error tolerance of 0.1, an alpha error of 0.05, and a design effect of 2, at least 192 health professionals were needed, and 224 health professionals were eventually recruited.

### Instruments

Two questionnaires were designed based on local policies and relevant research. Some experts who were experienced in HHCSs, including geriatricians, general practitioners and community nurses, were invited to help revise the questionnaires. Two pilot studies were conducted in the same population in *Haidian* district using the first edition of the questionnaires to ensure the practicability of the questionnaires 1 month before the formal investigations.

#### Questionnaire for the caregivers (Additional file 1)

The questionnaire for the caregivers was composed of demographic information of the older adults and the caregivers, the health status of the older adults, older adults’ disability level, and the caregivers’ demands for HHCSs.

Demographic information of the older adults such as sex, age, educational degree and health status of the older adults such as the cause of disability, medical history, use of assistive devices, skin problems, vision and hearing were collected through the caregiver. The disability level was measured using the Physical Self-Maintenance Scale (PSMS) and Instrumental Activities of Daily Living Scale (IADL) designed by Lawton & Brody. Each item had four options: “can do it independently”, “have some difficulties”, “need some help” and “cannot do it at all”, with responses ranging from 1 to 4. Activities scored 3 and 4 were defined as disabled. Older adults who had 1 or 2 disabled activities in PSMS were described as mildly disabled, those who had 3 or 4 disabled activities were moderately disabled, and those who had 5 or 6 disabled activities were severely disabled.

For the caregivers, in addition to basic information such as sex, age and educational degree, the relationship between the caregiver and the older adults, and the time spent caring for the older adults were also collected. Finally, the caregivers’ demands for HHCSs were investigated. Twenty-three items of HHCSs were divided into “home nursing services” and “health guidance services”. Home nursing services included specimen collection, defecation assistance, expectoration assistance, home oxygen therapy, intramuscular/subcutaneous injection, catheter management, indwelling needle management, pressure ulcer management, gastrointestinal intubation management, measurements of blood pressure/blood glucose/electrocardiogram, venous infusion and wound dressing/stitch removal. Health guidance services included chronic pain management, knowledge of chronic diseases (hypertension, diabetes, coronary disease, and cerebral stroke), knowledge of common diseases (except for chronic diseases), guidance on the caring ability of caregivers, guidance on drug use, guidance on home safety, guidance on lifestyle, guidance on domestic medical device operation, guidance on domestic rehabilitation device operation, guidance on rehabilitation training and psychological counselling. The caregiver’s demand score (CDS) of every item of HHCSs was measured using a 5-point Likert-type scale, ranging from “do not need it at all” to “need it very much”.

#### Questionnaire for health professionals (Additional file 2)

The questionnaire for the health professionals included personal information and the professional’s attitudes towards HHCSs. Personal information consisted of sex, age, job and years working in the CHSI, income, and physical and mental health statuses. The health professionals’ attitude score (HPAS) for the 23 items of HHCSs was graded on a 5-point Likert-type scale with responses ranging from 1 to 5 points, which represented “very unreasonable” to “very reasonable”. Finally, the professionals were all asked to choose three problems of the HHCSs out of nine choices in order of importance.

Cronbach’s α values of the CDS and HPAS were 0.950 and 0.959, respectively, indicating good internal consistency. Principal component analysis was conducted to confirm the factorability of CDS (KMO = 0.912, Bartlett’s test of sphericity *P* < 0.001) and HPAS (KMO = 0.950, Bartlett’s test of sphericity *P* < 0.001). The results of HPAS revealed 2 factors. One was composed of 12 items representing home nursing services, and the other was composed of 11 items representing health guidance services. The cumulative contribution rate was 70.423%. The results of CDS revealed 4 factors. The first one comprised 12 items representing home nursing services, and the other 11 items in the other 3 factors belonged to health guidance services. The cumulative contribution rate was 71.636%. The questionnaires had good construct validity.

### Data collection

Data from caregivers were collected by paper questionnaires using face-to-face interviews. Trained investigators asked the caregivers about the items on the questionnaire and completed the questionnaires for the caregivers. Then, they examined the questionnaires for logic errors and missing information to ensure that the questionnaires were completed correctly and carefully. Caregivers were allowed to leave only if no logic errors or missing information were present in the questionnaire. The investigation of caregivers was conducted from May to September 2018.

Web-based questionnaires were used by health professionals. The directors of the CHSIs were contacted before the survey to confirm the number of qualified health professionals. Then, they shared the link for the questionnaire using WeChat to all the health professionals in the selected CHSIs who met all of the aforementioned criteria. A minimum time limit was established to ensure the completeness of the questionnaires. If there was any blank on the questionnaire, the questionnaire could not be submitted. The questionnaires from a CHSI were required to be submitted within a week; otherwise, the research team asked the director for help. The investigation of health professionals was conducted from August to September 2018.

### Statistical analysis

The data were entered into IBM Statistical Package for Social Science version 19.0 (SPSS Inc., Chicago IL, US) for analysis after checking by the research team. The study variables are described as means with standard deviations (*SD*) and percentages. The Kolmogorov-Smirnov test was used to assess the normality of HPAS and CDS data for HHCSs. T-tests and Wilcoxon signed-rank tests were used to detect the differences between the caregivers’ and community health professionals’ views on HHCSs.

## Results

### Demographic data of the older adults and caregivers

Ten of the 380 recruited caregivers declined to participate in the survey, and thus the response rate was 97.4%. These 370 caregivers completed the questionnaires, and all these questionnaires were valid.

The age of disabled older adults ranged from 61 to 101 years (*mean* = 80.9 years, *SD* = 8.4 years), and 64.9% of them were aged over 80 years. Most of the older people lived with their family members (83.8%). Approximately 70% of the older people had more than three diseases simultaneously. The primary source of income for these older adults was mostly from their own pension (73.5%), and over half of them had medical insurance for urban workers (59.2%). Details are shown in Table 1.

**Table 1:** Demographic data and some health status of the disabled older adults (*N* = 370)

Characteristics	N (%)
*Sex*	
Male	180 (48.6)
Female	190 (51.4)
*Age*	
60–69 years	44 (11.9)
70–79 years	86 (23.2)
80 years and older	240 (64.9)
*Marriage*	
Married	209 (56.5)
Divorced/separated	2 (0.5)
Widowed	158 (42.7)
Unmarried	1 (0.3)
*Medical insurance*	
Free medical treatment	73 (19.7)
Medical insurance for urban workers	219 (59.2)
Medical insurance for urban residents	78 (21.2)
*Education*	
Bachelor’s degree or above	97 (26.2)
Middle school	155 (41.9)
Primary school or below	118 (31.9)
*Primary income source*	
Own pension	272 (73.5)
Supported by family members	75 (20.3)
House rent or other investment	18 (4.9)
Government assistance	4 (1.1)
Other	1 (0.3)
*Current monthly income (yuan)*	
2000 and below	61 (16.5)
2001–4000	98 (26.5)
4001–6000	119 (32.2)
6001–8000	39 (10.5)
8001 and above	53 (14.3)
*Living conditions*	
Living alone	1 (0.3)
Living only with family members	233 (63.0)
Living only with hired caregivers	59 (15.9)
Living with family members and hired caregivers	77 (20.8)
*Number of chronic diseases*	
1	30 (8.1)
2	84 (22.7)
3 or more	256 (69.2)
*Bed-ridden*	
Yes	66 (17.8)
No	304 (82.2)
*Communication*	
Able	312 (84.3)
Unable	58 (15.7)
*Disability level*	
Mild	169 (45.7)
Moderate	77 (20.8)
Severe	124 (33.5)

The caregivers were aged from 21 to 90 years (*mean* = 58.0 years, *SD* = 11.6 years), and 68.9% were female. Most were family members of the older adults (70.8%). Less than half of the families hired a domestic caregiver (45.4%). Most caregivers had cared for older adults for more than 16 h per day (67.0%), and > 80% of them cared for older adults for less than 10 years. Details are shown in Table 2.

**Table 2:** Demographic data of the caregivers (*N* = 370)

Characteristics	N (%)
*Sex*	
Male	115 (31.1)
Female	255 (68.9)
*Age*	
40 years and younger	12 (3.2)
41–49 years	74 (20.0)
50–59 years	138 (37.3)
60–69 years	85 (23.0)
70 years and older	61 (16.5)
*Education*	
Bachelor’s degree or above	84 (22.7)
Middle school	217 (58.7)
Primary school or below	69 (18.6)
*Relationship*	
Hired	108 (29.2)
Family member	262 (70.8)
*Hired a domestic*	
Yes	168 (45.4)
No	202 (54.6)
*Salary for the hired caregivers*	
3000 yuan or less	8 (4.8)
3001–6000 yuan	156 (92.8)
6001 yuan or more	4 (2.4)
*Number of co-caregivers*	
0	152 (41.4)
1	75 (20.3)
2	87 (23.5)
3 or more	56 (15.1)
*Years of caring*	
Less than 1 year	32 (8.6)
1–10 years	269 (72.7)
11–20 years	64 (17.3)
21 years or more	5 (1.4)
*Hours of care provided per day*	
3–8 h	68 (18.4)
9–15 h	54 (14.6)
16 h or more	248 (67.0)

### Caregivers’ willingness to apply for HHCSs for older adults

Of the 370 caregivers, 314 (84.9%) would like to accept HHCSs for the older adults they provided care, but only 20.5% (*N* = 76) received HHCSs. Over 80% of the caregivers would like to pay less than 100 yuan as an out-of-pocket expense for HHCSs per visit (Table 3).

**Table 3:** The out-of-pocket expenses the caregivers would like to pay for HHCSs per visit (*N* = 314)

Out-of-pocket expenses for HHCSs per visit	N (%)
50 yuan or less	121 (38.5)
51–100 yuan	132 (42.1)
101–200 yuan	47 (15.0)
201–300 yuan	8 (2.5)
301 yuan or more	6 (1.9)

Fifty-six caregivers (15.1%) were unwilling to accept HHCSs. The top three reasons for their unwillingness were “worry about the high out-of-pocket expenses (69.6%)”, “the expenses may not be covered by insurance (41.1%)” and “do not know the content of the HHCSs (40.4%)” (Table 4).

**Table 4:** Reasons for caregivers’ unwillingness to accept HHCSs (multiple choice, *N* = 56)

Reasons	N (%)
Worry about the high out-of-pocket expenses	39 (69.6)
The expenses may not be covered by insurance	23 (41.1)
Do not understand the content of the HHCSs	21 (40.4)
The content of the service is limited	14 (37.5)
Worry about privacy	14 (25.0)
Do not want to be disturbed	12 (21.4)
Distrust of community health care workers	9 (16.1)

### Demographic data and health status of the health professionals

Of the 224 questionnaires administered the health professionals, all were recalled and valid. The health professionals were mostly aged 36–45 years (46.4%), and women accounted for 80.8%. The majority had received a bachelor’s degree (66.1%), and 56.3% of them had a middle title. GPs (43.8%) and community nurses (29.0%) made up the majority of the participants. Most health professionals earned 5001–10,000 yuan per month (68.3%), while the expected salary was mainly 10,001–20,000 yuan (67.8%). Over 40% of the participants considered their physical and mental health status “good” or “very good” (Table 5).

**Table 5:** Demographic data and health status of the health professionals (*N* = 224)

Characteristics	N (%)
*Sex*	
Male	43 (19.2)
Female	181 (80.8)
*Age*	
25 years and younger	8 (3.6)
26–35 years	67 (29.9)
36–45 years	104 (46.4)
46–55 years	39 (17.4)
56 years and older	6 (2.7)
*Work experience*	
Less than 5 years	56 (25.0)
5–10 years	69 (30.8)
11–15 years	63 (24.1)
16 years or more	36 (16.1)
*Job*	
General practitioner	98 (43.8)
Community nurse	65 (29.0)
Public health doctor	11 (4.9)
TCM physician, psychological consultant or rehabilitation therapist	21 (9.4)
Other	29 (13.0)
*Professional title*	
Junior title	56 (25.0)
Middle title	127 (56.3)
Senior title	20 (8.9)
Other	21 (9.4)
*Education*	
Master’s degree or above	20 (8.9)
Bachelor’s degree	148 (66.1)
Junior college or below	56 (25.0)
*Current monthly income*	
5000 yuan and lower	60 (26.8)
5001–10,000 yuan	153 (68.3)
10,001 yuan and higher	11 (4.9)
*Expected monthly income*	
5001–10,000 yuan	29 (13.0)
10,001–15,000 yuan	72 (32.1)
15,001–20,000 yuan	80 (35.7)
20,001 yuan and higher	43 (19.2)
*Physical health status*	
Very good	41 (18.3)
Good	97 (43.3)
Moderate	81 (36.2)
Poor	3 (1.3)
Very poor	2 (0.9)
*Mental health status*	
Very good	58 (25.9)
Good	94 (42.0)
Moderate	65 (29.0)
Poor	4 (1.8)
Very poor	3 (1.3)

### Problems associated with providing the HHCSs selected by the health professionals

Table 6 shows the problems health professionals selected when providing HHCSs. The top three chosen problems were “health professionals’ personal safety cannot be guaranteed” (67.4%), “lack of health professionals to fulfil the HHCSs” (58.0%) and “lack of laws and regulations governing HHCSs” (46.0%).

**Table 6:** Problems selected by health professionals when providing the HHCSs (multiple choice, *N* = 224)

Problems	Total N (%)
Health professionals’ personal safety cannot be guaranteed	151 (67.4)
Lack of health professionals	130 (58.0)
Lack of laws and regulations	103 (46.0)
The medical risk	91 (40.6)
Low charge for HHCSs does not reflect the work value	78 (34.8)
Lack of operational standards	70 (31.3)
Lack of necessary equipment for travelling, diagnosing and recording	26 (11.6)
Lack of incentive mechanism	18 (8.0)
Waste of medical resources	7 (3.1)

### Health professionals’ attitudes and caregivers’ demands

The five most reasonable items rated by the health professionals were “guidance on drug use (4.12)”, “knowledge of common diseases (4.10)”, “knowledge of chronic diseases (4.09)”, “guidance on lifestyle (4.05)” and “guidance on home safety (4.04)”. All of these items belonged to the category of “health guidance services”. The five most unreasonable items were “venous infusion (2.82)”, “defecation assistance (3.11)”, “indwelling needle management (3.20)”, “stomach tube management (3.29)” and “wound dressing/stitch removal (3.37)”. All five items belonged to the category of “home nursing services”.

The top five demands of caregivers were “test of blood pressure, blood glucose or electrocardiogram (4.33)”, “specimen collection (4.21)”, “intramuscular/subcutaneous injection (4.19)”, “venous infusion (4.16)” and “guidance on drug use (3.76)”. Four of them belonged to the category of “home nursing services”. The bottom five demands of caregivers were “psychological counselling (3.09)”, “guidance on domestic medical device operation (3.14)”, “knowledge of common diseases (3.28)”, “guidance on lifestyle (3.30)” and “indwelling needle management (3.34)”. Four of them belonged to the category of “health guidance services”.

The Wilcoxon signed-rank test was used to detect the differences because of the nonnormality of the HPAS for health guidance services and CDS for home nursing services. For the health professionals, the HPAS for home nursing services (*mean* = 3.39, *SD* = 0.84) was significantly lower (*Z* = − 10.081, *P* < 0.001) than the score for health guidance services (*mean* = 4.00, *SD* = 0.70), and the overall mean score was 3.68 (*SD* = 0.69). For the caregivers, the CDS for home nursing services (*mean* = 3.76, *SD* = 0.86) was significantly higher (*Z* = − 7.725, *P* < 0.001) than the score for health guidance services (*mean* = 3.43, *SD* = 0.82), and the overall mean score was 3.60 (*SD* = 0.74). A significant difference was not observed between overall HPAS and CDS using the *t*-test (*t* = 1.341, *P* = 0.180). For home nursing services, the HPAS was lower than the CDS (*Z* = -4.960, *P* < 0.001), while for health guidance services, the HPAS was higher than the CDS (*Z* = -8.373, *P* < 0.001). Table 7 shows health professionals’ and caregivers’ views on every item of HHCSs.

**Table 7:** Health professionals’ and caregivers’ views on HHCSs

Home Health Care Service	Health professionals’ attitudes towards HHCSs (mean ± SD)^**a**^	Caregivers demands for HHCSs for the disabled older adults (mean ± SD)^**b**^
***Home nursing***		
Blood pressure/blood glucose/electrocardiogram testing	3.92 ± 1.06	4.33 ± 0.91
Catheter management	3.48 ± 1.03	3.74 ± 1.21
Defecation assistance	3.10 ± 1.15	3.44 ± 1.16
Expectoration assistance	3.39 ± 1.04	3.49 ± 1.14
Gastrointestinal intubation management	3.30 ± 1.10	3.72 ± 1.18
Home oxygen therapy	3.57 ± 1.02	3.58 ± 1.10
Indwelling needle management	3.21 ± 1.07	3.34 ± 1.19
Intramuscular/subcutaneous injection	3.43 ± 1.16	4.19 ± 0.99
Specimen collection	3.46 ± 1.06	4.21 ± 0.95
Ulcer management	3.70 ± 1.00	3.57 ± 1.24
Venous infusion	2.79 ± 1.40	4.16 ± 1.00
Wound dressing/stitch removal	3.38 ± 1.08	3.37 ± 1.20
***Health guidance***		
Chronic pain management	3.86 ± 0.84	3.42 ± 1.11
Domestic medical device operation guidance	3.94 ± 0.87	3.14 ± 1.05
Domestic rehabilitation device operation guidance	3.99 ± 0.79	3.62 ± 1.08
Drug use guidance	4.13 ± 0.78	3.76 ± 0.96
Guidance on the caring ability of caregivers	3.98 ± 0.80	3.41 ± 1.03
Home safety guidance	4.03 ± 0.83	3.35 ± 1.06
Knowledge of chronic diseases	4.10 ± 0.81	3.69 ± 0.97
Knowledge of common diseases	4.11 ± 0.79	3.28 ± 1.07
Lifestyle guidance	4.04 ± 0.88	3.30 ± 1.02
Psychological counselling	3.96 ± 0.83	3.09 ± 1.03
Rehabilitation method guidance	3.99 ± 0.82	3.70 ± 1.04

^a^Health professionals’ attitudes towards HHCS were measured by 5-point Likert-type scale with responses ranging from 1 to 5 points representing “very unreasonable” to “very reasonable”

^b^Caregivers demands for HHCS for the disabled older adults were measured by 5-point Likert-type scale with responses ranging from 1 to 5 points representing “do not need it at all” to “need it very much”

## Discussion

We aimed to detect potential differences between community health professionals’ and caregivers’ views on HHCSs for disabled older adults. The results might reveal their distinct views.

### Caregivers’ unmet needs for HHCSs may be relevant to insufficient accessibility

Over 80% of the investigated caregivers would like to apply for HHCSs for disabled older adults, but only 20.5% of the older adults had ever received HHCSs. The unmet demands for HHCSs may be due to the insufficient supply of health professionals and insufficient accessibility for disabled older people and their caregivers. Usually, CHSIs appoint at least one experienced health professional to provide HHCSs per visit. However, compared with seeing and treating outpatients in CHSIs, providing HHCSs will require more time per patient, which appears to be inefficient. Therefore, health professionals tend to avoid introducing or providing these services to residents and make HHCSs less accessible. To some extent, an unmet need for information on HHCSs affects the utilization of HHCSs by caregivers, consistent with a previous study conducted in America [28].

In the present study, over 80% of the caregivers wanted to pay less than 100 yuan as an out-of-pocket expense for HHCSs per visit. “Worry about the high out-of-pocket expenses” and “the expenses may not be covered by insurance” were the main reasons why caregivers were unwilling to accept HHCSs. Based on the results, some measures, e.g., encouraging older adults to buy commercial insurance and/or LTCI, should be implemented to reduce the out-of-pocket of HHCSs costs in Beijing. Previous studies showed that the HHCSs system would run smoothly if LTCI was developed [29, 30].

### Health professionals’ problems in delivering HHCSs

Most health professionals chose “health professionals’ personal safety cannot be guaranteed” as an important problem. Some previous studies reported that health professionals sometimes had to endure verbal abuse during home visits [31, 32], and worries about personal safety may decrease enthusiasm to deliver HHCSs. Practitioners may feel frustrated and helplessness when experiencing problems such as sexual harassment and requests to perform tasks beyond their duties because assistance was not available [33]. Limited advocacy for health professionals’ rights was one of the barriers to providing HHCSs. The mature experience of some CHSIs is worth learning. During the process of providing HHCSs to *Fangzhuan*g CHSIs located in *Fengtai* District, Beijing, the staff of the subdistrict office/neighbourhood committee witnessed the whole implementation process of HHCSs and could promote doctor-patient communication and protect personal safety for health professionals [34].

The first diagnosis and treatment in CHSIs as one of the four core contents of the hierarchical diagnosis and treatment policy was implemented nationwide in 2015 [35]. Since then, CHSIs were considered as the first choice by more and more community residents with mild symptoms. In 2018, compared with the average daily outpatient visits recorded by doctors in general hospitals of 9.1, the average daily outpatient visits recorded by doctors in CHSIs were 18.1 (centres) and 20.8 (stations) in Beijing [36], which indicated health professionals’ heavy workload in CHSIs. In our study, 58% of health professionals chose “lack of health professionals to fulfil the HHCSs” as the secondary problem in providing HHCSs. Due to the limited staffing in CHSIs, HHCSs cannot be effectively popularized and can only be provided for some elderly individuals with severe problems or specific needs.

“Lack of laws and regulations governing HHCSs” was chosen as the third important problem in delivering HHCSs. The result was consistent with previous qualitative research of our research team in Beijing, which also indicated that the lack of sound regulations potentially restricted the development of HHCSs [37]. It is noteworthy that the national policy on HHCSs namely *“Notice on Strengthening Home Health Care Services for Older Adults”* was promulgated in December 2020. It consisted of five parts, “Elements of HHCSs”, “Standardize the practice of HHCSs”, “Strengthen the management of HHCSs”, “Strengthen support and guarantee for HHCSs” and “Organization and implementation” [38]. HHCSs have drawn attention at the national level. Provincial and municipal governments should embark on local policies of HHCSs in accordance with national policies to promote the development of HHCSs.

### Different views on HHCSs between health professionals and caregivers

The results for the CDS of caregivers and HPAS of health professionals revealed differences. The CDS for “home nursing services” was significantly higher than the score for “health guidance services”. This result indicated that caregivers had urgent needs for professional nursing care rather than health guidance services, as related knowledge can be obtained from books, brochure, the internet, and other forms of social media [39]. In our study, health professionals would rather provide “health guidance services” than “home nursing services”. Two factors might explain the differences. First, given the lack of medical equipment and restricted sanitary conditions in the older adults’ homes [40], most health professionals prefer to perform high risk operations, e.g., venous infusion, in CHSIs rather than the patients’ homes. Usually, non-invasive operations, e.g., health guidance and blood pressure measurement, and low risk invasive HHCSs, e.g., intramuscular/subcutaneous injection, were operated at patients’ homes. Second, the relatively low educational level and clinical competence deficiency of health professionals in CHSIs undeniably influence the implementation of HHCSs to some degree. In 2018, 48.4% of physicians and 21% of nurses in CHSIs had a bachelor’s degree or higher, compared with 71.7% of physicians and 23.7% of nurses in second- and third-tier hospitals in China [36]. Additionally, compared with health professionals for HHCSs in America and Japan, who are required to train for 2 to 3 months in the home-based setting and work with experienced health professionals during home visits, the vast majority of health professionals performing HHCSs in China are not trained or well trained, accompanied by a lack of confidence in providing HHCSs [32].

### Strengths and limitations

Previous studies focused on HHCSs from the perspective of either caregivers or health professionals, while the present study integrated the views of caregivers and health professionals, enabling a direct comparison of the underlying differences and conflicts between them.

Our work mainly focused on determining the differences between the views of caregivers and health professionals towards HHCSs, but caregivers’ characteristics, such as age and health status, may affect their willingness to receive HHCSs, and many factors can influence health professionals’ attitudes towards HHCSs as well. We plan to examine the factors that influence caregivers’ and health professionals’ views on HHCSs in the future. In addition, we chose the ADL to screen disabled older adults, and a few older adults with only mental disabilities may have been missed. Finally, the sample was recruited from only one district, and randomization could not be accomplished due to the absence of a list of disabled older adults in Beijing; therefore, the results had limited representativeness. The conditions in different populations should be detected in the future.

## Conclusions

The unmet demands for HHCSs may be due to the insufficient supply of health professionals and insufficient accessibility for disabled older people and their caregivers. Certain gaps in views on HHCSs were identified between health professionals and caregivers. Compared to health professionals with a higher willingness to provide health guidance services, caregivers prefer to receive home nursing services. Personal safety, an insufficient number of health professionals, and the lack of laws and regulations governing HHCSs influence the provision of HHCSs.

Based on these results, policy makers should implement feasible policies and measures to increase the availability of HHCSs, including establishing and implementing policies to safeguard rights and interests of health professionals and training qualified health professionals for HHCSs. In the future, more comprehensive studies should be conducted to improve the implementation of HHCSs for older adults.

## Supplementary Information

**Additional file 1.** Questionnaire for caregivers on the views of home health care services for the disabled older adults.

**Additional file 2.** Questionnaire for health professionals on the views of providing home health care services for the disabled older adults.

## Data Availability

The datasets generated and analyzed during the current study are not publicly available to protect participant privacy, but are available from the corresponding author on reasonable request.

## Abbreviations

HHCSs: Home health care services
CHSIs: Community health service institutions
GPs: General practitioners
ADL: Activities of Daily Living
LTC: Long-term care
LTCI: Long-term care insurance
TCM: Traditional Chinese medicine
PSMS: Physical Self-Maintenance Scale
IADL: Instrumental Activities of Daily Living Scale
CDS: Caregiver’s demand score
HPAS: Health professionals’ attitude score
SD: Standard deviation
