# Supplementary material for: Comparison of the caregivers’ and community health professionals’ views on home health care services for disabled older adults: a cross-sectional study in Beijing, China
Source: BMC Health Serv Res. 2021 Apr 26;21:389. doi: 10.1186/s12913-021-06400-9 (PMC8077684; doi:10.1186/s12913-021-06400-9)
Supplement: Supplementary file 1 — Additional file 1. Questionnaire for caregivers on the views of home health care services for the disabled older adults. [file 12913_2021_6400_MOESM1_ESM.pdf]

Questionnaire for Caregivers on The Views of Home Health Care Services for The Disabled Older Adults

Demographic information of the caregiver

1.Sex:

(1) □ Male (2) □ Female

2. Age:______

3. What's your highest education qualification?

(1) □ Bachelor degree or above (2) □ Middle school (3) □ Primary school or below

4.What's your relationship with the older adult?

(1) □ Spouse (2) □ Children (3) □ Other family members (4) □ Hired, the salary is _____ yuan per month.

5.If you are a family member of the older adult, did you hire someone to take care of him/her?

(1) □No (2) □Yes, the salary is ______ yuan per month.

6. Are there any other caregivers?

(1) □No (2) □Yes, there are _____ caregivers in total.

7. How many years have you taken care of the older adult?

_____years

8. How many hours do you take care of the older adult every day?

_____hours

Demographic information of the disabled older adult

1. Sex:

(1) □ Male (2) □ Female

2. Age:______

3. Highest education qualification:

(1) □ Bachelor degree or above (2) □ Middle school (3) □ Primary school or below

4. Marriage

(1) □ Married (2) □ Divorced/separated (3) □ Widowed (4) □ Unmarried

5. Living conditions (multiple choice) □

(1) □ Living alone (2) □ Living with spouse (3) □ Living with children (4) □ Living with hired caregivers (5) □ Living with another person

6. Personal monthly income:

(1) □ 2000 yuan and below (2) □ 2001-4000 yuan (3) □ 4001-6000 yuan (4) □ 6001-8000 yuan (5) □ 8001 yuan and above

7. Source of income:

(1) □ Own pension (2) □ Supported by family members (3) □ House rent or other investment (4) □ Government assistance (5) □ Other________

8. Medical insurance:

(1) □ Free medical treatment (2) □ Medical insurance for urban workers (3) □ Medical insurance for urban residents

Health status of the disabled older adult

9. Chronic diseases (multiple choice):

(1) □ Hypertension (2) □ Diabetes (3) □ Coronary heart disease (4) □ Cerebrovascular disease (5) □ Osteoarthritis (6) □ Osteoporosis (7) □ Dementia (8) □ Malignant tumor (9) □ Chronic lung disease (10) □ Chronic gastrointestinal diseases (11) □ Prostate disease (12) □ Other__________

10. Bed-ridden:

(1) □ Yes (turn to 11) (2) □ No (turn to 12)

11. Pressure sores:

(1) □ No (2) □ Yes, _______cm

12. Has he/she fallen down in the past year?

(1) □ No (2) □Yes, because______________________

13.Communication:

(1) □ Able (2) □ Unable

14.Pain:

(1) □ No (2) □ Yes, at_________________

15. Vision:

(1) □ Normal (2) □ Decreased but not affecting life (3) □ Decreased and affecting life (4) □ Blind (single eye or double eyes)

16. Hearing:

(1) □ Normal (2) □ Decreased but not affecting life (3) □ Decreased and affecting life (4) □ Deaf (single ear or double ears)

17. Urination:

(1) □ Normal (2) □ Uracratia (3) □ Uroschesis (4) □ Other condition_________

18. Defecation:

(1) □ Normal (2) □ Fetal incontinence (3) □ Constipation (4) □ Other condition_________

Disability level of the older adult

19.Activity of Daily Living Scale (ADL):

Items	Can do it independently	Have some difficulties	Need some help	Can’t do it at all
Use of transportation				
Locomotion				
Meal preparation				
Housekeeping				
Responsibility for medication intake				
Feeding				
Dressing				

Grooming				
Laundry				
Bathing				
Shopping				
Toileting				
Telephone use				
Handling finances				

The demands for home health care services (HHCSs)

20. Are you willing to accept the HHCSs provided by the community health service center for the disabled older adult?

(1) □ Yes (turn to 22) (2) □No (turn to 21)

21. Why don’t you want to apply HHCSs for the older adult (multiple choice):

(1) □ Worry about the high out-of-pocket expenses

(2) □ The expenses may not be covered by insurance

(3) □ Do not understand the content of the HHCSs

(4) □ The content of the service is limited

(5) □ Worry about privacy

(6) □ Do not want to be disturbed

(7) □ Distrust of community health care workers

22. How much can you accept per visit?

(1) □ 50 yuan and below (2) □ 51-100 yuan (3) □ 101-200 yuan (4) □ 201-300 yuan (5) □ 301 yuan and above

23. Demands on every item of HHCSs

Categories	Items	Need it very much	Need it	Moderate	Don’t really need it	Don’t need it at all
Home nursing services	Blood pressure/blood glucose/electrocardiogram testing	5☐	4☐	3☐	2☐	1☐
	Catheter management	5☐	4☐	3☐	2☐	1☐
	Defecation assistance	5☐	4☐	3☐	2☐	1☐
	Expectoration assistance	5☐	4☐	3☐	2☐	1☐
	Gastrointestinal intubation management	5☐	4☐	3☐	2☐	1☐
	Home oxygen therapy	5☐	4☐	3☐	2☐	1☐
	Indwelling needle management	5☐	4☐	3☐	2☐	1☐
	Intramuscular/subcutaneous injection	5☐	4☐	3☐	2☐	1☐
	Specimen collection	5☐	4☐	3☐	2☐	1☐

	Ulcer management	5☐	4☐	3☐	2☐	1☐
	Venous infusion	5☐	4☐	3☐	2☐	1☐
	Wound dressing/stitch removal	5☐	4☐	3☐	2☐	1☐
Health guidance services	Chronic pain management	5☐	4☐	3☐	2☐	1☐
	Domestic medical device operation guidance	5☐	4☐	3☐	2☐	1☐
	Domestic rehabilitation device operation guidance	5☐	4☐	3☐	2☐	1☐
	Drug use guidance	5☐	4☐	3☐	2☐	1☐
	Guidance on the caring ability of caregivers	5☐	4☐	3☐	2☐	1☐
	Home safety guidance	5☐	4☐	3☐	2☐	1☐
	Knowledge of chronic diseases	5☐	4☐	3☐	2☐	1☐
	Knowledge of common diseases	5☐	4☐	3☐	2☐	1☐
	Lifestyle guidance	5☐	4☐	3☐	2☐	1☐
	Psychological counselling	5☐	4☐	3☐	2☐	1☐
	Rehabilitation method guidance	5☐	4☐	3☐	2☐	1☐
