# Supplementary material for: Comparison of the caregivers’ and community health professionals’ views on home health care services for disabled older adults: a cross-sectional study in Beijing, China
Source: BMC Health Serv Res. 2021 Apr 26;21:389. doi: 10.1186/s12913-021-06400-9 (PMC8077684; doi:10.1186/s12913-021-06400-9)
Supplement: Supplementary file 2 — Additional file 2. Questionnaire for health professionals on the views of providing home health care services for the disabled older adults. [file 12913_2021_6400_MOESM2_ESM.pdf]

Questionnaire for Health Professionals on The Views of Providing Home Health Care Services for The Disabled Older Adults

Demographic and health information

1.Sex:

(1) □ Male (2) □ Female

2. Age:______

3. How many years have you worked in this community health service center?

______years.

4. What's your job in the community health service center?

(1) □ General practitioner (2) □ Community nurse (3) □ Public health doctor (4) □ TCM physician, psychological consultant or rehabilitation therapist (5) □ Other______

5. What's your professional title?

(1) □ Junior title (2) □ Middle title (3) □ Senior title (4) □ Other

6. What's your highest education qualification?

(1) □ Master degree or above (2) □ Bachelor degree (3) □ Junior college or below

7. What's your monthly salary over the past year, including basic salary, bonus and other income?

(1) □ 5000 yuan and below (2) □ 5001-10000 yuan (3) □10001 yuan and above

8. What's your expected monthly salary, including basic salary, bonus and other income?

(1) □ 5001-10000 yuan (2) □ 10001-15000 yuan (3) □ 15001-20000 yuan (4) □ 20001 yuan and above

9. What's your health condition?

(1) □ Very good (2) □ Good (3) □ Moderate (4) □ Poor (5) □ Very poor

10. What's your mental health condition?

(1) □ Very good (2) □ Good (3) □ Moderate (4) □ Poor (5) □ Very poor

Problems in carrying out home health care services (HHCSs)

11. What are the top three problems in carrying out HHCSs in your opinion? ____________

A. Health professionals' personal safety cannot be guaranteed
B. Lack of health professionals
C. Lack of laws and regulations
D. The medical risk
E. Low charge for HHCSs does not reflect the work value
F. Lack of operational standards
G. Lack of necessary equipment for travelling, diagnosing and recording
H. Lack of incentive mechanism
I. Waste of medical resources

Attitudes on HHCSs

12. Attitudes on every item of HHCSs

Categories	Items	Very reasonable	Reasonable	Moderate	Unreasonable	Very unreasonable	I don't care

Home nursing services	Blood pressure/blood glucose/electrocardiogram testing	5□	4□	3□	2□	1□	0□
	Catheter management	5□	4□	3□	2□	1□	0□
	Defecation assistance	5□	4□	3□	2□	1□	0□
	Expectoration assistance	5□	4□	3□	2□	1□	0□
	Gastrointestinal intubation management	5□	4□	3□	2□	1□	0□
	Home oxygen therapy	5□	4□	3□	2□	1□	0□
	Indwelling needle management	5□	4□	3□	2□	1□	0□
	Intramuscular/subcutaneous injection	5□	4□	3□	2□	1□	0□
	Specimen collection	5□	4□	3□	2□	1□	0□
	Ulcer management	5□	4□	3□	2□	1□	0□
	Venous infusion	5□	4□	3□	2□	1□	0□
	Wound dressing/stitch removal	5□	4□	3□	2□	1□	0□
Health guidance	Chronic pain management	5□	4□	3□	2□	1□	0□

services	Domestic medical device operation guidance	5☐	4☐	3☐	2☐	1☐	0☐
	Domestic rehabilitation device operation guidance	5☐	4☐	3☐	2☐	1☐	0☐
	Drug use guidance	5☐	4☐	3☐	2☐	1☐	0☐
	Guidance on the caring ability of caregivers	5☐	4☐	3☐	2☐	1☐	0☐
	Home safety guidance	5☐	4☐	3☐	2☐	1☐	0☐
	Knowledge of chronic diseases	5☐	4☐	3☐	2☐	1☐	0☐
	Knowledge of common diseases	5☐	4☐	3☐	2☐	1☐	0☐
	Lifestyle guidance	5☐	4☐	3☐	2☐	1☐	0☐
	Psychological counselling	5☐	4☐	3☐	2☐	1☐	0☐
	Rehabilitation method guidance	5☐	4☐	3☐	2☐	1☐	0☐
